# Vitamin D Status in Adolescents during COVID-19 Pandemic: A Cross-Sectional Comparative Study

**DOI:** 10.3390/nu13051467

**Published:** 2021-04-26

**Authors:** Martina Meoli, Franco Muggli, Sebastiano A.G. Lava, Mario G. Bianchetti, Carlo Agostoni, Claudine Kocher, Thomas W. Bührer, Letizia Ciliberti, Giacomo D. Simonetti, Gregorio P. Milani

**Affiliations:** 1 Facoltà di Scienze Biomediche, Università della Svizzera Italiana, 6900 Lugano, Switzerland; martina.meoli93@gmail.com (M.M.); fmuggli@bluewin.ch (F.M.); mario.bianchetti@usi.ch (M.G.B.); giacomo.simonetti@eoc.ch (G.D.S.); 2 Pediatric Cardiology Unit, Department of Pediatrics, Centre Hospitalier Universitaire Vaudois, University of Lausanne, 1011 Lausanne, Switzerland; webmaster@sebastianolava.ch; 3 Pediatric Unit, Fondazione IRCCS Ca’ Granda Ospedale Maggiore Policlinico, via della Commenda 9, 20122 Milan, Italy; carlo.agostoni@unimi.it (C.A.); letizia.ciliberti@unimi.it (L.C.); 4 Department of Clinical Sciences and Community Health, Università degli Studi di Milano, 20122 Milan, Italy; 5 Swiss Department of Defence, Medical Intelligence, 3000 Bern, Switzerland; Claudine.Kocher@vtg.admin.ch (C.K.); Thomas.Buehrer@vtg.admin.ch (T.W.B.); 6 Pediatric Institute of Southern Switzerland, Ospedale San Giovanni, 6500 Bellinzona, Switzerland

**Keywords:** lockdown, complications, adolescents, vitamins, diet, insufficiency, pandemic, COVID19, SARS-CoV-2, prevention

## Abstract

Vitamin D has been claimed to be effective in the response to infections, including the respiratory syndrome coronavirus 2 (SARS-CoV-2). It is supposed that lockdown measures and fear of SARS-CoV-2 infection might reduce vitamin D levels through the modification of lifestyle. However, very few data exist on the association between lockdown measures and vitamin D status in humans. For this cross-sectional comparative study, adolescents (*n* = 298) aged 18 to 19 years were enrolled during the compulsory military fitness-for-duty evaluation between July and December 2020 in Southern Switzerland. Beyond anthropometric measurements, participants filled in a structured questionnaire about their lifestyle and a blood specimen was sampled for the determination of total 25-hydroxy-vitamin D. The obtained data were compared with those of 437 adolescents enrolled at the military fitness-for-duty evaluation during the same period of the year in the context of the CENERI study (2014–2016). The anthropometric measures were similar between the two study groups. The levels of vitamin D were also comparable (77 (64–91) vs. 74 (60–92) nmol/L, *p* = 0.50; median and interquartile range). A total of 38 (13%) and 43 (9.8%) subjects presented insufficient (<50 nmol/L) levels of vitamin D (*p* = 0.42) during the current pandemic and in the CENERI study, respectively. These data do not support the hypothesis that during the SARS-CoV-2 pandemic, late adolescents are at higher risk of vitamin insufficiency.

## 1. Introduction

Vitamin D has been claimed to be effective in modulating the response to bacterial and viral diseases, including the coronavirus disease 19 (COVID-19) [1,2].

In many western countries, including Switzerland, individuals have been instructed to practice social and physical distancing in both indoor and outdoor spaces by maintaining a minimum distance from other people outside their household. Closing of unnecessary stores, schools and indoor recreation facilities and the use of home working, curfews and quarantines have also been recommended.

Since sunlight exposure is the main factor affecting vitamin D status, it is speculated that the mentioned measures might negatively impact the vitamin D status of the general population [3]. The pandemic has also modified other aspects of lifestyle [4], such as physical activity and the consumption of tobacco, which, in turn, might affect the vitamin D metabolism [5,6]. However, very few data exist on the association between lockdown measures and vitamin D and available data are mainly from adult subjects and the elderly [7,8]. The aim of this study was to investigate whether the current pandemic had an impact on vitamin D levels in male late adolescents residing in Southern Switzerland, a region with high solar radiation.

## 2. Materials and Methods

For the purpose of this cross-sectional comparative study, we enrolled subjects 18 to 19 years of age during the physical examination for the compulsory military service between July and December 2020 in Southern Switzerland. Eligible subjects were all male, without any chronic or acute condition or treatment potentially affecting the level of circulating vitamin D, such as chronic liver or kidney diseases, management with corticosteroids or antiepileptics. Subjects declaring supplementation with vitamin D were also excluded. Body height, weight and waist circumference were collected according to a standardized protocol [9]. Body height and weight were used to calculate the body mass index. A non-stretching tape was used to measure waist circumference at the iliac crest. Sitting arterial blood pressure and heart rate were measured three times in subjects in resting conditions for at least 5 min with a validated Microlife^®^ oscillometric monitor [10]. The average value of the three measurements was then calculated. In the same morning of the above-mentioned measurements, subjects underwent blood sampling for the determination of total 25-hydroxy-vitamin D as previously described [9,11]. Briefly, an Abbott chemiluminescent immunoassay, whose accuracy and reliability are monitored both in the Vitamin D External Quality Assessment Scheme and in the Vitamin D Standardization Program [12,13], was used to measure both 25-hydroxy vitamin D_2_ and 25-hydroxy vitamin D_3_. Finally, enrolled subjects filled in a structured questionnaire investigating the current frequency of recreational physical activity (never, 1 per week, 2–4 per week, 5–6 per week, every day) and smoking behavior (never, 1–10 cigarettes/day, 11–20 cigarettes/day, >20 cigarettes/day). The 2020 data were compared with those obtained in 437 adolescents enrolled with the same inclusion and exclusion criteria during the same period of the year in the context of the CENERI study (2014-2016) [11]. The levels of total 25-hydroxy-vitamin D were considered adequate if ≥50 nmol/L or insufficient if <50 nmol/L [9]. Data are presented as median and interquartile range, as box and whiskers plot, or as frequency and percentage. The Mann–Whitney U test was used to compare continuous values and the χ^2^ test to compare categorical data. The Bonferroni adjustment was used to reduce the type I error when performing multiple statistical tests on vitamin D levels.

GraphPadPrism 9.02 (GraphPad Software, San Diego, CA, USA) was used for statistical analyses.

## 3. Results

Among the 900 adolescents who underwent the medical examination from July to December 2020, 298 (30%) volunteered to participate. Subjects who participated in the study did not differ from the other subjects in terms of body height, weight and waist circumference (data not shown).

Body height, weight, body mass index and waist circumference were similar in the two study groups, i.e., in the period 2014–2016 and in July–December 2020 (Table 1). The arterial systolic and diastolic blood pressure and the heart rate were also comparable in the two study periods.

**Figure 1:**
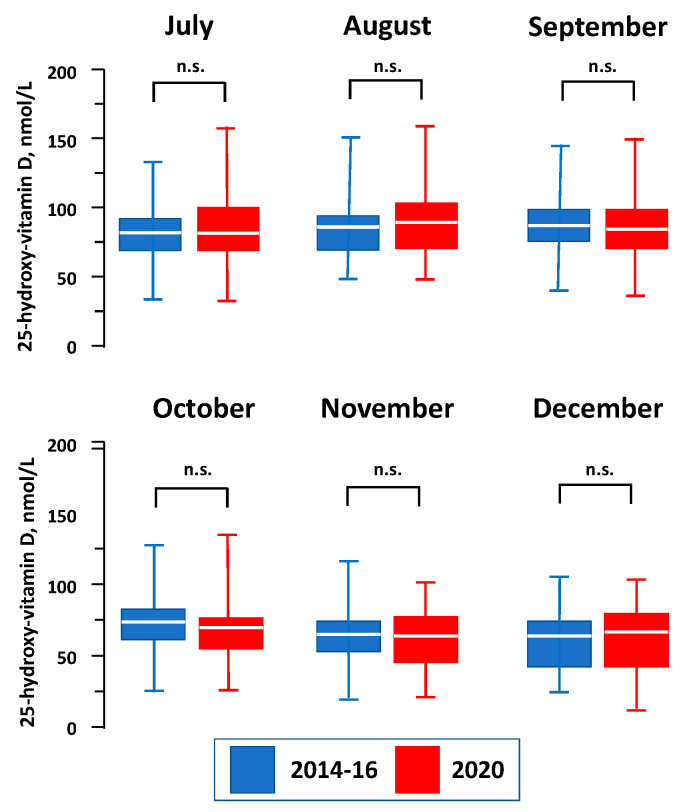
Levels of 25-hydroxy-vitamin D across the months of the study periods. Boxes represent the 25th and 75th percentile, the white line the median and the whiskers the minimum and maximum levels of 25-hydroxy-vitamin D. The Mann–Whitney U Test with the Bonferroni adjustment was used. n.s. = not significant.

The levels of the total 25-hydroxy-vitamin D were not different in 2014–2016 as compared with the 2020 study period (77 (64–91) vs. 74 (60–92) nmol/L, *p* = 0.50). The levels were also similar across the months (*p* = ns for all the comparisons): 81 (69–92) vs. 82 (67–102) nmol/L in July, 82 (69–92) vs. 88 (72–106) nmol/L in August, 81 (73–97) vs. 76 (63–97) nmol/L in September, 73 (60–84) vs. 65 (53–74) nmol/L in October, 63 (51–74) vs. 60 (42–78) nmol/L in November and 61 (43–73) vs. 65 (42–81) nmol/L in December (Figure 1). A total of 43 (9.8%) and 38 (13%) presented insufficient levels of vitamin D (*p* = 0.42) in 2014–2016 and in 2020, respectively (Table 1). Vitamin D_2_ levels were ≤5 nmol/L in all subjects.

The frequency of subjects who never performed recreational physical activity was higher in 2014–2016 as compared to 2020 (99 (23%) vs. 26 (8.7%), *p* < 0.001). Similarly, the overall frequency of recreational physical activity was higher (*p* = 0.02) in 2020 as compared with 2014–2016. Regarding smoking attitudes, the number of non-smoker subjects (249, (57%), vs. 170 (57%), *p* = 0.08) was similar between the two study periods, but among smokers, a tendency to consume more cigarettes was observed in 2020 as compared to 2014–2016 (Table 1). 

## 4. Discussion

Distancing and cessation of usual activities might impact several aspects of health, ranging from changes in lifestyle to mood disorders [3,14]. Therefore, it is widely assumed that home confinement measures adopted to counteract the spread of severe acute respiratory syndrome coronavirus 2 (SARS-CoV-2) may be associated with consequences on human metabolism, including vitamin D production secondary to sun exposure [15,16]. The present cross-sectional comparative study does not support such assumptions in male late adolescents of Southern Switzerland.

The results of this study are in line with a retrospective study comparing the values of vitamin D measured in a laboratory hospital from 2018 to 2020 in adult subjects [7]. The authors of the study found that the percentage of adults with vitamin D levels < 50 nmol/L was approximately 18% in the 2018–2019 period and 16% in 2020. The reasons underlying these observations were not investigated in detail either in the mentioned study or in our investigation. However, some hypotheses can be formulated. In the last few months of pandemic, the risk of vitamin D insufficiency has been emphasized by scientific societies and media in European countries and it is possible that the population made efforts to increase sunlight exposure during the lockdown [3]. This hypothesis is supported by the finding that the frequency of subjects performing recreational physical activity was higher in 2020 as compared with the previous years. Considering that, due to lockdown measures, indoor sport centers (e.g., gyms or swimming pools) were closed, it is possible to assume that adolescents spent more time outdoors for physical activities.

A few data have suggested that cigarette consumption increased during lockdown [17]. This finding might be relevant for vitamin D metabolism, since cotinine, an alkaloid of tobacco**,** negatively affects circulating vitamin D levels in youth [18]. We did not observe an increase in the number of smokers among late adolescents enrolled in 2020, but a tendency to smoke more cigarettes among smokers. These findings further suggest that other factors, such those mentioned above associated with physical activity, were more determinant in the metabolism of vitamin D. However, new research investigating the behavioral changes associated with vitamin D metabolism is needed to confirm this hypothesis.

Although this study did not identify any difference in vitamin D levels before and during the pandemic, the results confirm that insufficient levels of vitamin D are detectable in more than 10% of adolescents even in non-winter seasons and that achieving adequate levels of circulating vitamin D in this age group is still a major challenge for public health interventions [19,20]. It is well recognized that the bone mass peak is achieved in males from 25 to 35 years of life and is strongly associated with vitamin D status [21]. Therefore, late adolescence is one of the most critical phases for the development of bone mass and a poor vitamin D status might have detrimental consequences later in life for skeletal health [22].

In the context of the current pandemic, growing data suggest that inadequate levels of vitamin D are also associated with an increased risk of SARS-CoV-2 infection in young subjects [1,23]. On the other hand, the administration of vitamin D has no beneficial effect on patients with COVID-19 [24,25]. Given that vitamin D levels may act as a negative acute phase reactant, studies on this topic and future interventions should consider all these potential factors to prevent misinterpretation of clinical data and accurately assess the role of vitamin D status in patients with COVID-19 [26].

The present data should be viewed in light of some considerations. We did not have data on vitamin D levels from January to June or in female subjects, and longitudinal changes in vitamin D levels were not investigated, which are the main limitations of the study. However, comparisons of each month in the two study periods 2014–2016 versus 2020 did not detect any significant difference. We did not distinguish outdoor from indoor recreational activity and did not collect data on diet (and especially on food rich in vitamin D) and occult sources of vitamin D such as “restorative medicines”, which are likely very popular during this pandemic. Recent data from different countries showed that the increased time available for cooking modified the dietary habits of adolescents, favoring the consumption of foods such as vegetables [27]. However, available data do not suggest a significant change in overall diet quality [27]. The main strength of this study is that it prospectively involved an unselected sample of male subjects aged between 18 and 19 years. Since medical evaluation before the military service is compulsory for all male adolescents in many countries, including Switzerland, it represents a valuable source of information for the health status of the general population of male late adolescents [28,29].

## 5. Conclusions

In conclusions, this study does not support the hypothesis that, during the current pandemic, late adolescents are at higher risk of vitamin D insufficiency as compared to previous years.

## Figures and Tables

**Table 1:** Characteristics of the late adolescents included in the CENERI study (2014–2016) and in 2020 *.

	2014–2016	2020	*p*-Value
***n***	437	298	
**Body weight**, kg	72.5 (65.7–84.0)	72 (65.0–80.0)	0.99
**Body height**, m	1.78 (1.73–1.82)	1.77 (1.73–1.81)	0.66
**Body mass index**, kg/m^2^	23.0 (21.0–25.1)	22.4 (20.9–25.3)	0.50
**Waist circumference**, cm	81 (75–88)	80 (75–87)	0.06
**Blood pressure**, mmHg			
Systolic	132 (123–136)	125 (122–135)	0.12
Diastolic	76 (70–82)	73 (69–80)	0.47
**Heart rate**, beats/minute	72 (66–84)	71 (61–82)	0.64
**Frequency of recreational physical activity**			0.02
Never	101 (23%)	26 (8.7%)	
1 per week	75 (17%)	86 (29%)	
2–4 per week	185 (42%)	150 (50%)	
5–6 per week	41 (8.7%)	27 (9.1%)	
Every day	35 (10%)	9 (3.0%)	
**Smoking**			0.03
Never	249 (57)	170 (57)	
1–10 cigarettes/day	126 (29)	63 (21)	
11–20 cigarettes/day	67 (15)	64 (21)	
>20 cigarettes/day	5 (1.1)	1 (0.3)	
**Total 25-hydroxy-vitamin D, nmol/L**	77 (64–91)	74 (60–92)	0.50

* Data are given as median and interquartile range or frequency and percentage. The Mann–Whitney U test or the χ^2^ test were applied, as appropriate.

## Data Availability

Data are available upon reasonable request.
